# miRNA-122-5p stimulates the proliferation and DNA synthesis and inhibits the early apoptosis of human spermatogonial stem cells by targeting CBL and competing with lncRNA CASC7

**DOI:** 10.18632/aging.104158

**Published:** 2020-11-24

**Authors:** Fan Zhou, Wei Chen, Yinghong Cui, Bang Liu, Qingqing Yuan, Zheng Li, Zuping He

**Affiliations:** 1 Hunan Normal University School of Medicine, Changsha 410013, Hunan, China; 2 State Key Laboratory of Oncogenes and Related Genes, Renji-Med X Clinical Stem Cell Research Center, Department of Plastic Surgery, Ren Ji Hospital, School of Medicine, Shanghai Jiao Tong University, Shanghai 200127, China; 3 Department of Andrology, Urologic Medical Center, Shanghai General Hospital, Shanghai Jiao Tong University, Shanghai 200080, China; 4 The Key Laboratory of Model Animals and Stem Cell Biology in Hunan Province, Changsha 410013, Hunan, China

**Keywords:** miRNA-122-5p, human spermatogonial stem cells, proliferation and early apoptosis, transcription factor CBL, lncRNA CASC7

## Abstract

Epigenetic regulators of human spermatogonia stem cells (SSCs) remain largely unknown. We found that miRNA-122-5p was upregulated in human spermatogonia from obstructive azoospermia (OA) patients compared with non-obstructive azoospermia (NOA). MiRNA-122-5p stimulated the proliferation and DNA synthesis of human SSCs, whereas it inhibited the early apoptosis of human SSCs. CBL was predicted and identified as a direct target of miRNA-122-5p in human SSCs. CBL silencing led to an enhancement of cell proliferation and DNA synthesis and neutralized the effect of miRNA-122-5p inhibitor on the DNA synthesis of human SSCs. The decrease in the early apoptosis of human SSCs was observed after CBL knockdown. By comparing the profiles of lncRNAs between OA and NOA spermatogonia, CASC7 was significantly deficient in OA spermatogonia, and it had a direct association with miRNA-122-5p. LncRNA CASC7 competed with miRNA-122-5p, and it suppressed the inhibition of CBL. Collectively, these results implicate that miRNA-122-5p enhances the proliferation and DNA synthesis and inhibits the early apoptosis of human SSCs by targeting CBL and competing with lncRNA CASC7. Therefore, this study provides novel insights into epigenetic regulation of fate determinations of human SSCs, and it offers new targets for gene therapy of male infertility that is associated with aging.

## INTRODUCTION

Spermatogonial stem cells (SSCs) can produce mature and functional spermatids through the precise process, namely spermatogenesis, and male germ cells from SSCs to spermatids are able to transmit genetic information across generations [1]. Notably, SSCs can be reprogramed to obtain pluripotency, dedifferentiate to become embryonic stem-like (ES-like) cells [2], and directly transdifferentiate to functional cells of other lineages [3, 4] and tissues [5]. As such, SSCs could be utilized in reproductive and regenerative medicine.

In the testis, SSCs undergo self-renewal to maintain stemness of the cells, and they differentiate to spermatocytes and mature spermatids [6]. The self-renewal and differentiation of SSCs are precisely regulated by epigenetic factors and genetic elements [7–10]. Non-coding RNAs, including microRNAs (miRNAs) and long non-coding RNAs (lncRNAs), are one of the most significant epigenetic factors that determine the fate decisions of the cells, and they perform biological modifications without changing DNA sequences. MiRNAs are endogenous RNA molecules with a length of 18-25 nucleotides (nt) [11, 12], while lncRNAs are transcribed RNA molecules with a length of over 200 nt [13, 14]. MiRNAs complementarily bind to the sequences in the 3’ UTRs of the targeting mRNAs, which results in the degradation of mRNA or translation inhibition [15]. LncRNAs diversely control gene expression, and it has been suggested that lncRNAs interact with miRNAs by acting as endogenous sponges or competing endogenous RNA (ceRNA), which further affects the expression of targeting genes [16–19].

MiRNAs play important roles in regulating the self-renewal, differentiation, and apoptosis of SSCs. It has been shown that miRNA-221/222 is essential for maintaining the undifferentiated state of mammalian spermatogonia through the repression of KIT expression [20]. We have presviously demonstrated that miRNA-20 and miRNA-106a enhance the division of mouse SSCs throught targeting STAT3 and Ccnd1 [21], while Chen et al. [22] have revealed that miRNA-202 retains the stemness of mouse SSCs by targeting cell cycle regulators and RNA binding proteins. Moreover, miRNA-100 and miRNA-10b stimulate mouse SSC proliferation by regulating Stat3 and Kruppel-like factor 4, respectively [23, 24]. Chd1l-miRNA-486-MMP2 has been shown to be a regulatory axis for the stemness and growth property of SSCs [25], and miRNA-322 is essential for SSC self-renewal by targeting RASSF8 (ras association domain family 8) [26]. MiRNA-17-92 cluster and miRNA-290-295 cluster control the proliferation and/or differentiation of SSCs [27, 28]. Tong *et al.* [29] have revealed that miRNA-let7 family miRNAs are required for spermatogonial differentiation induced by RA. Deletion of Mir-17-92 cluster in mice leads to an increase in the level of miRNA-106b-25, reflecting the functional cooperation of these two miRNA clusters [30]. MiRNA-146 modulates mouse SSC differentiation by the regulation of RA [31]. MiRNA-34c controls the differentiation of mouse SSCs via targeting Nanos2 [32], and miRNA-17-92 is essential for normal spermatogenesis in mice [33].

Additionally, lncRNAs emerge as the novel determinants of stem cell self-renewal and differentiation [34, 35]. LncRNA033862 has been suggested to be crucial for the maintenance of SSC proliferation and survival by regulating *Gfrα1* [36], while lncRNA AK015322 promotes the proliferation of mouse SSCs and serves as a decoy of miRNA-19b-3p [37]. LncRNA Mrhl negatively regulates the expression of *Sox8* and it is important for spermatogonial differentiation [38]. Recently, lncRNA Gm2044 has been shown to inhibit the proliferation of mouse spermatogonia [39].

To date, very little is known about the interaction of miRNAs and lncRNAs in human SSCs. We have previously identified that miRNA-663a is important for the proliferation and DNA synthesis and suppresses the early apoptosis of human SSCs by targeting transcription factor NFIX [40]. It is interesting to unveil the networks among lncRNAs, miRNAs, and their targets in human SSCs. In this study, we have explored the expression, function, and targets of miRNA-122-5p in human SSCs, and we have demonstrated that miRNA-122-5p stimulates the proliferation and DNA synthesis and inhibits the early apoptosis of human SSCs line by targeting CBL. Furthermore, we have found that lncRNA CASC7 competes with the miRNA-122-5p and controls the level of transcription factor CBL. This study on miRNA-122-5p and its functional axis significantly offers novel insights into the epigenetic regulation of human SSCs, and it provides the scientific basis on molecular therapy for treating male infertility.

## RESULTS

### Isolation and identification of human spermatogonia from OA and NOA patients’ testicular tissues

Human male germ cells were isolated from the testicular tissues of obstructive azoospermia (OA) and non-obstructive azoospermia (NOA) patients using a two-step enzymatic digestion and followed by differential plating. Human spermatogonia were further separated by STA-PUT velocity sedimentation by 0.5-4% BSA from OA and NOA patients [41]. Morphological features and phenotypic identification of human spermatogonia were conducted by our previous work [40].

### Differentially expressed MiRNAs in human spermatogonia between OA and NOA patients

**Figure 1:**
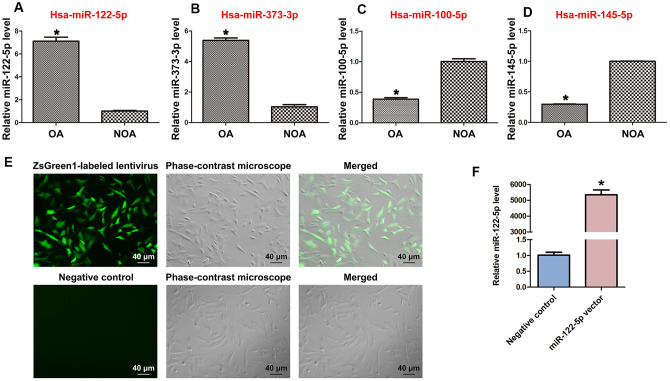
**Differentially expressed MiRNAs of human spermatogonia between OA and NOA patients and transfection efficiency of ZsGreen1-labeled lentivirus in human SSC line.** (**A–D**) Real-time qPCR revealed the different expression levels of miRNA-122-5p (miR-122-5p) (**A**), miR-373-3p (**B**), miR-100-5p (**C**), and miR-145-5p (**D**) in human spermatogonia between OA and NOA patients. * indicates statistically significant differences (p< 0.05) in human spermatogonia between human OA and NOA patients. (**E**) Fluorescence microscope and phase-contrast microscope indicated the transfection efficiency of ZsGreen1-labeled miRNA-122-5p lentivirus compared to the control lentivirus of human SSC line. Scale bars =40 μm. (**F**) Real-time qPCR showed the relative levels of miRNA-122-5p in human SSCs treated with miRNA-122-5p vector and control lentivirus. * indicated statistically significant differences (p< 0.05) in human SSC line between miRNA-122-5p vector and the control lentivirus.

MiRNA microarrays were utilized by us to compare global miRNA profiles in human spermatogonia between OA and NOA patients [42]. Among the differentially expressed miRNAs, miRNA-122-5p was expressed at a higher level in human spermatogonia of OA patients compared to NOA patients, which was verified by our real-time qPCR (Figure 1A). These data indicate that miRNA-122-5p plays a potential role in regulating the fate determinations of human SSCs. In addition, we observed that miR-373-3p was expressed at a higher level in human spermatogonia of OA patients than NOA patients (Figure 1B), whereas the levels of miR-100-5p and miR-145-5p were lower in human spermatogonia of OA patients compared with NOA patients (Figure 1C, 1D).

### Phenotypic identification and overexpression of MiRNA-122-5p in human SSC line

Human SSC line has been established by us, and phenotypic characteristics of this cell line were verified as SSCs in our recent work [40]. Human SSC line was infected lentivirus with miRNA-122-5p sequences, and as shown by ZsGreen1-labeled miRNA-122-5p lentivirus, the infection efficiency of miRNA-122-5p lentivirus in the human SSC line was over 80% (Figure 1E). After transfection for 24 hr, real-time qPCR revealed that the expression level of miRNA-122-5p in human SSC line was significantly upregulated by miRNA-122-5p lentivirus compared to control lentivirus (Figure 1F).

### MiRNA-122-5p stimulates the proliferation and DNA synthesis and decreases the early apoptosis of human SSCs

**Figure 2:**
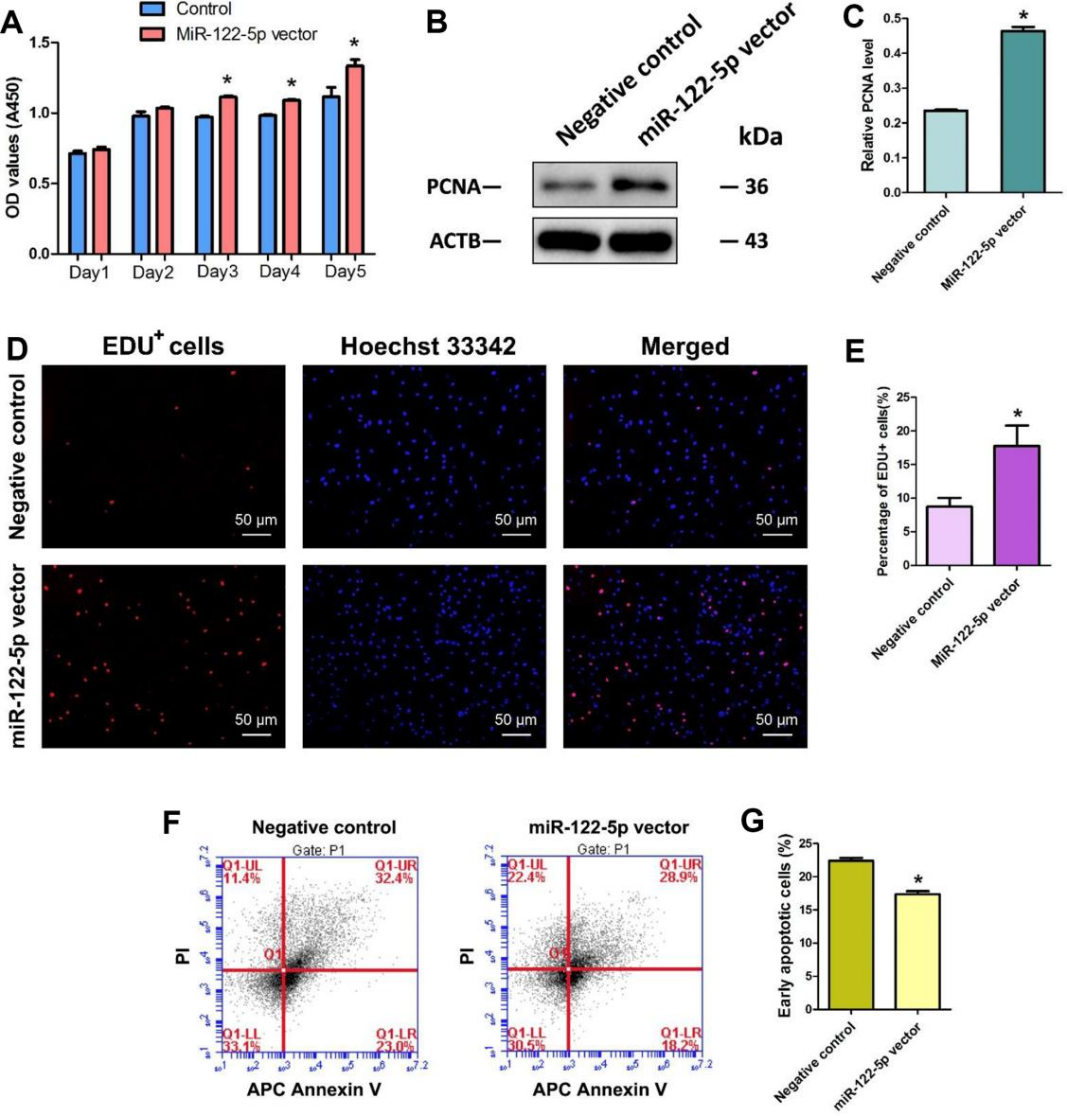
**Effect of overexpression of MiRNA-122-5p on the proliferation, DNA synthesis and early apoptosis of human SSC line.** (**A**) CCK-8 assays revealed the growth curve of human SSC line treated with miRNA-122-5p vector and control lentivirus in 5 days. (**B**) Western blots demonstrated PCNA expression in human SSC line of miRNA-122-5p vector and control lentivirus. (**C**) The relative expression of PCNA in human SSC line of miRNA-122-5p vector and control lentivirus after normalization to the signals of their loading control ACTB. (**D**) EDU incorporation assay showed the EDU-positive cells in human SSC line of miRNA-122-5p vector and control lentivirus. Scale bars =50 μm. (**E**) Qualification of EDU-positive cells in human SSC line of miRNA-122-5p vector and control lentivirus. (**F**, **G**) APC Annexin V and PI staining and flow cytometry showed the percentages of early apoptosis in human SSC line infected with ZsGreen1-labeled miRNA-122-5p lentivirus and control lentivirus. * denoted statistically significant differences (p< 0.05) in human SSC line between control lentivirus and miRNA-122-5p vector.

We determined the influence of miRNA-122-5p on the division of human SSC line using several methods. Cell proliferation assays revealed that the proliferation of human SSC line was obviously increased by miRNA-122-5p lentivirus compared to control lentivirus at day 3 to day 5 of culture (Figure 2A). Western blots demonstrated that level of PCNA protein was enhanced in human SSC line at day 5 by miRNA-122-5p lentivirus vs. the control lentivirus (Figure 2B, 2C). Moreover, EDU incorporation assay indicated that the percentage of EDU-positive cells was increased in human SSC line at day 5 by miRNA-122-5p lentivirus vector (Figure 2D, 2E). Collectively, these results suggest that miRNA-122-5p stimulates the division and DNA synthesis of human SSCs. Annexin V and propidium iodide (PI) staining and flow cytometry assays showed that miRNA-122-5p lentivirus reduced the early apoptosis but not the late apoptosis of human SSC line (Figure 2F, 2G), implicating that miRNA-122-5p inhibits the early apoptosis of human SSCs.

### CBL is a direct target for MiRNA-122-5p in human SSC line

**Figure 3:**
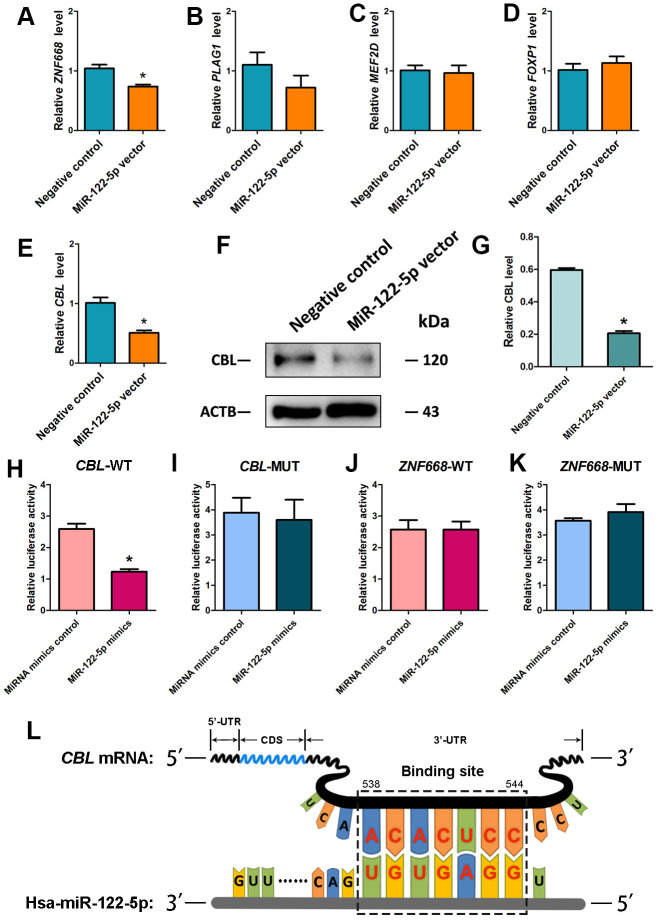
**Identification and verification of the target CBL of MiRNA-122-5p in human SSC line.** (**A–E**) Real-time qPCR revealed the relative levels of *ZNF668* (**A**), *PLAG1* (**B**), *MEF2D* (**C**), *FOXP1* (**D**) and *CBL* (**E**) in human SSCs of miRNA-122-5p vector and control lentivirus. * indicated statistically significant differences (p< 0.05) in human SSC line between control lentivirus and miRNA-122-5p vector. (**F**) Western blots depicted CBL protein in human SSC line of miRNA-122-5p vector and control lentivirus. (**G**) The relative expression levels of CBL protein in human SSC line of miRNA-122-5p vector and control lentivirus after normalization to the signals of their loading control ACTB. * denoted statistically significant differences (p< 0.05) in human SSC line between control lentivirus and miRNA-122-5p vector. (**H**, **I**) Validation of the targeting of miRNA-122-5p to wild type *CBL* and mutated *CBL* by dual luciferase reporter assays. (**J**, **K**) Validation of the targeting of miRNA-122-5p to wild type *ZNF668* and mutated *ZNF668* by dual luciferase reporter assays. * implied statistically significant differences (p< 0.05) in human SSC line between miRNA-122-5p mimics and miRNA mimics control. (**L**) Schematic diagram illustrated the binding site of miRNA-122-5p to *CBL* mRNA.

Using miRNA predict software, including Targetscan and miRDB, we predicted several potential targets of miR-122-5p. We verified these targets using real-time qPCR showing that the expression level of transcription factor *CBL* was downregulated by miRNA-122-5p lentivirus compared with other targets (Figures 3A–3E). Western blots further revealed that the level of CBL protein was significantly decreased by miRNA-122-5p lentivirus (Figure 3F, 3G). Therefore, CBL is one binding target of miRNA-122-5p in human SSCs. Furthermore, dual luciferase assays were performed to verify the binding cite of *CBL* mRNA. The predicted protein sequence in 3’UTR of *CBL* mRNA diminished the luciferase activity of the fusion genes in response to the treatment of miRNA-122-5p mimics (Figure 3H), whereas the mutated target sequence didn’t have any influence on the luciferase activity (Figure 3I). Neither the predicted target sequence in 3’UTR of *ZNF668* mRNA nor the mutated sequence had any effect on the luciferase activity (Figure 3J, 3K). Considered together, these data suggest that CBL is a direct target of miRNA-122-5p in human SSCs. The binding of seed region (from the 2^nd^ to 8^th^ nucleotides) on miRNA-122-5p to the target 3’UTR sequence of *CBL* mRNA was illustrated in Figure 3L.

### CBL silencing promotes the proliferation and inhibits the early apoptosis of human SSCs

**Figure 4:**
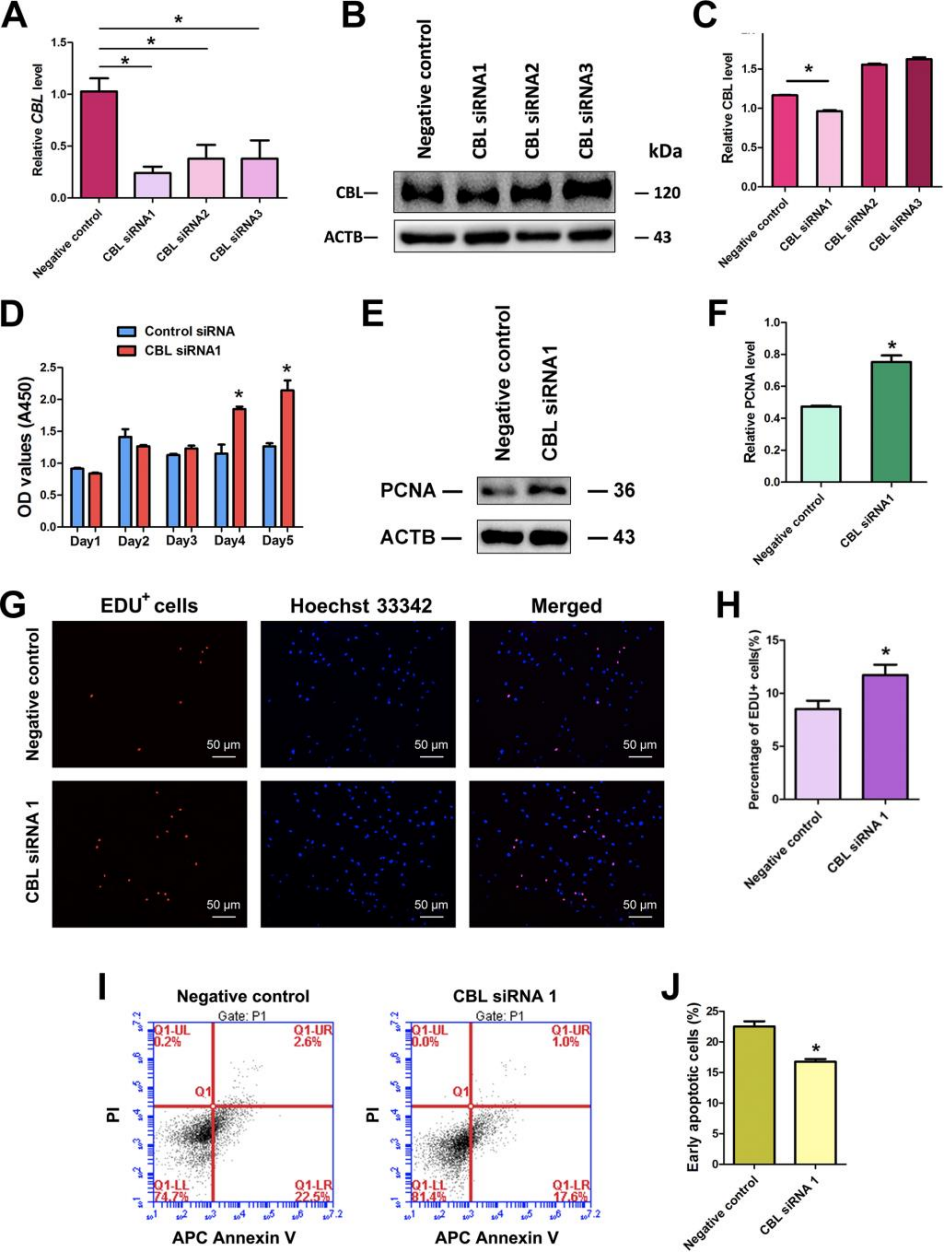
**Influence of CBL silencing on the proliferation, DNA synthesis, and early apoptosis of human SSC line.** (**A**) Real-time qPCR showed changes of *CBL* mRNA by CBL siRNA1, CBL siRNA2, and CBL siRNA3 in human SSC line. (**B**, **C**) Western blots revealed changes of CBL protein by CBL siRNA1, CBL siRNA2, and CBL siRNA3 in human SSC line. * denoted statistically significant differences (p< 0.05) of CBL siRNA1-, 2-, and 3-treated human SSC line compared with the control siRNA. (**D**) CCK-8 assays showed the growth curve of human SSC line treated with control siRNA and CBL siRNA1 for 5 days. * indicated statistically significant differences (p<0.05) in human SSC line between control siRNA and CBL siRNA1. (**E**) Western blots illustrated the changes of PCNA protein in human SSC line at day 3 after transfection of control siRNA and CBL siRNA1. (**F**) The relative protein level of PCNA in human SSC line at day 3 after transfection of control siRNA and CBL siRNA1 through normalization to the signals of their loading control ACTB. * showed statistically significant differences (p< 0.05) in human SSC line between control siRNA and CBL siRNA1. (**G**, **H**) EDU incorporation assay demonstrated the percentages of EDU-positive cells in human SSC line affected by control siRNA and CBL siRNA1. Scale bars= 50 μm. * indicated statistically significant differences (p<0.05) between CBL siRNA1-treated human SSC line and the control siRNA. (**I**, **J**) APC Annexin V and PI staining and flow cytometry depicted the percentages of early apoptosis in human SSC line transfected with control siRNA and CBL siRNA1. * displayed statistically significant differences (p<0.05) between CBL siRNA1-treated human SSC line and the control siRNA.

We further explored the effect of CBL on the fate decisions of human SSC line. We utilized three pairs of CBL siRNAs, namely CBL siRNA1, CBL siRNA2, and CBL siRNA3, with an aim to obtain sequence-specific siRNAs of CBL. The transfection efficiency of CBL siRNAs in human SSC line was more than 80%, as evidenced by the transfection of FAM-labeled fluorescent oligo [40]. Real-time qPCR showed that three CBL siRNAs reduced the *CBL* transcript, and notably, CBL siRNA1 had the highest level for *CBL* knockdown (Figure 4A). Western blots displayed that CBL siRNA1 significantly decreased the level of CBL protein (Figure 4B, 4C).

Proliferation assay showed that CBL siRNA1 enhanced the growth of human SSC line (Figure 4D), and PCNA protein level was increased in human SSC line at day 5 by CBL siRNA1 (Figure 4E, 4F). EDU incorporation assays demonstrated that the number of EDU-positive cells was obviously increased by CBL siRNA1 (Figure 4G, 4H). Annexin V and PI staining and flow cytometry assay indicated that CBL silencing led to the decrease in the early apoptosis of the human SSC line at day 3 (Figure 4I, 4J). Taken together, CBL knockdown stimulates the division and inhibits early apoptosis of human SSCs, which is consistent with the overexpression of miRNA-122-5p.

### Synergetic influence of MiRNA-122-5p and CBL on fate determinations of human SSC line

**Figure 5:**
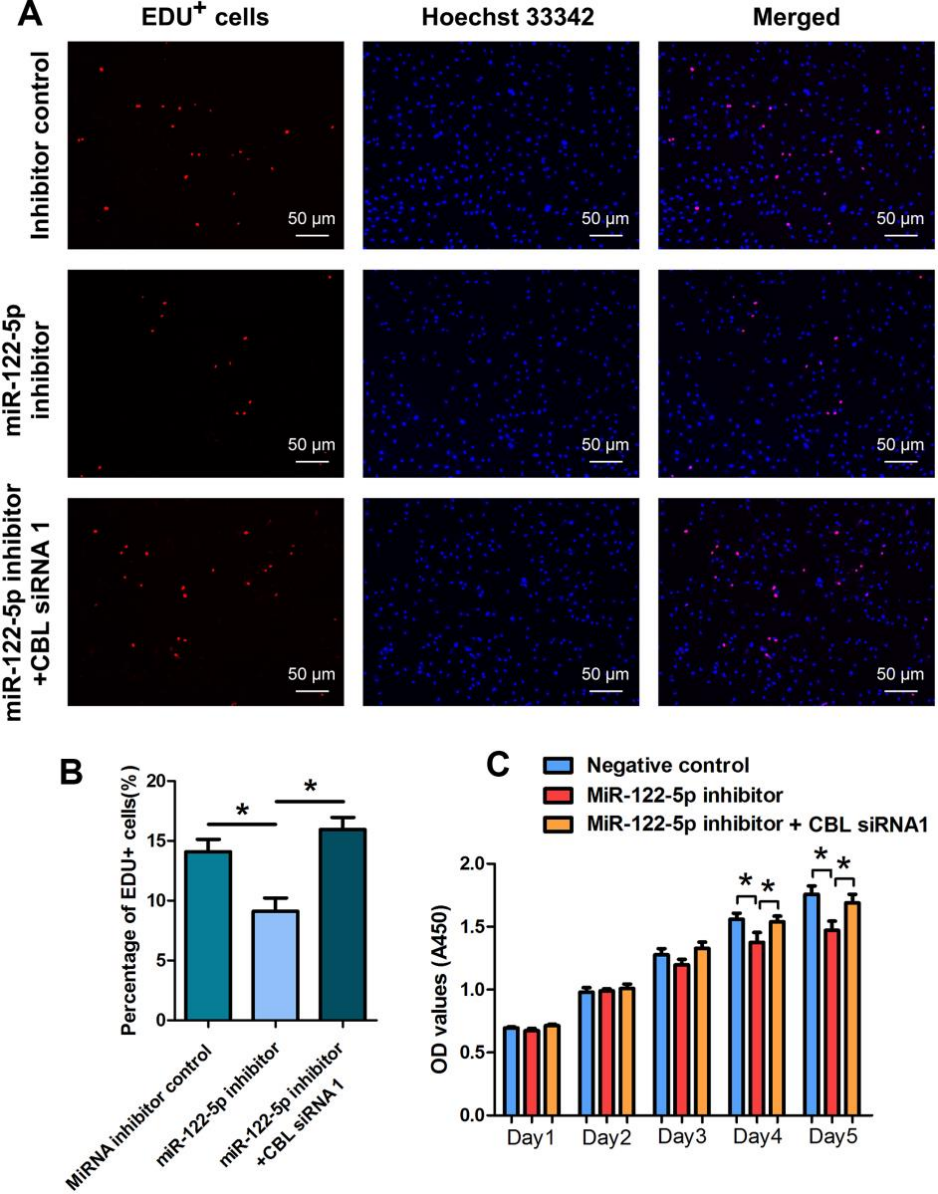
**The effect of MiRNA-122-5p inhibitor and CBL silencing on DNA synthesis and proliferation of human SSC line.** (**A**, **B**) EDU incorporation assay showed the percentages of EDU^+^ cells in human SSC line treated with miRNA inhibitor control, miRNA-122-5p inhibitor, as well as miRNA-122-5p inhibitor and CBL siRNA1. Scale bars = 50 μm. (**C**) CCK-8 assay showed the proliferation of human SSC line treated with miRNA inhibitor control, miRNA-122-5p inhibitor, and miRNA-122-5p inhibitor and CBL siRNA1 for 5 days. * denoted statistically significant differences in human SSC line (p< 0.05) between miRNA-122-5p inhibitor and the inhibitor control as well as the miRNA-122-5p inhibitor and miRNA-122-5p inhibitor plus CBL siRNA1.

We examined whether CBL had the synergetic effect with miRNA-122-5p on human SSC line using rescue assays. As shown in Figures 5A, 5B, the reduction in EDU-positive cells by miRNA-122-5p inhibitor was counteracted by CBL silencing in human SSC line. CCK-8 assays revealed that the proliferation decrease of human SSC line caused by miRNA-122-5p inhibitor was neutralized by CBL silencing at day 4 and day 5 (Figure 5C).

### LncRNA CASC7 is differentially expressed in human spermatogonia between OA and NOA patients

**Figure 6:**
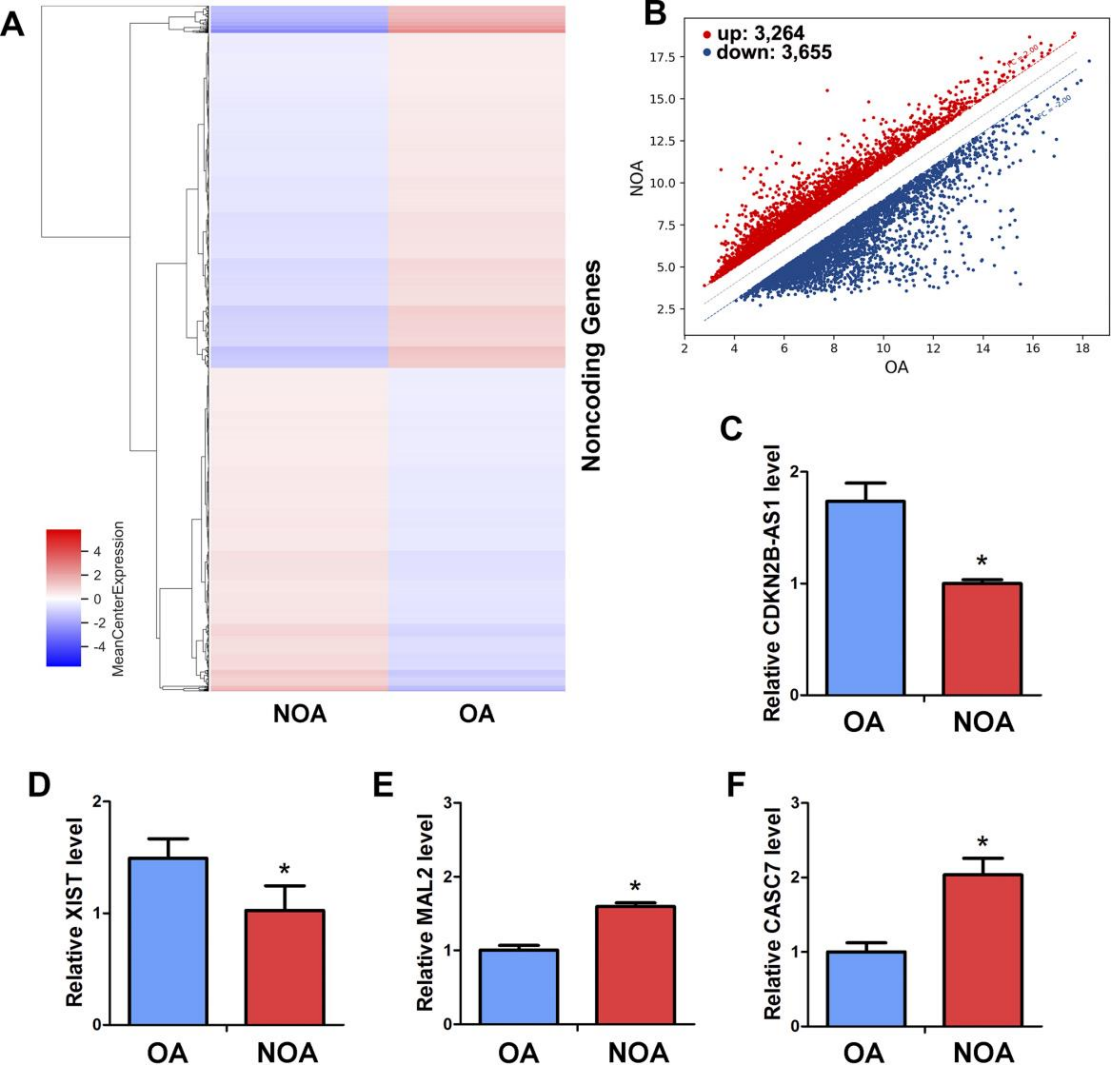
**Differentially expressed LncRNAs in human spermatogonia between OA and NOA patients.** (**A**) The cluster heat map showed lncRNAs with an expression change fold ≥2 from microarray data (p<0.025) in human spermatogonia from OA patients compared with NOA patients. (**B**) Scatter plot illustrated the differentially expressed lncRNAs with an expression change fold ≥2 (p<0.025) in human spermatogonia from OA patients compared with NOA patients. (**C–F**) Real-time qPCR revealed the different expression levels of XIST, CDKN2B-AS1, MAL2 and CASC7 in human spermatogonia between OA and NOA patients. * denoted statistically significant differences (p< 0.05) in human spermatogonia between human OA and NOA patients.

To further extend the molecular network of miRNA-122-5p in regulating the human SSCs, we compared and analyzed the profiles of lncRNAs in human spermatogonia between OA and NOA patients. As detected by an unbiased lncRNA microarray assay (Figure 6A), there were 6,919 lncRNAs whose expression levels were statistically significant (fold changes ≥ 2). In human spermatogonia between OA and NOA patients, 3,264 lncRNAs were upregulated whereas 3,655 lncRNAs were downregulated (Figure 6B). As predicted by Targetscan and miRDB software, we identified four lncRNAs that potentially had direct association with miRNA-122-5p from the differentially expressed lncRNAs in human spermatogonia between OA and NOA spermatogonia. These candidate lncRNAs were examined by real-time qPCR to validate the results of the microarray assay, and we found that CDKN2B-AS1 (Figure 6C) and XIST (Figure 6D) were expressed at higher levels in human spermatogonia of OA patients than NOA patients, whereas the levels of MAL2 (Figure 6E) and CASC7 (Figure 6F) were significantly lower in human spermatogonia of OA patients compared with NOA patients.

### LncRNA CASC7 competes with the MiRNA-122-5p and counteracts the inhibition of CBL in human SSCs

**Figure 7:**
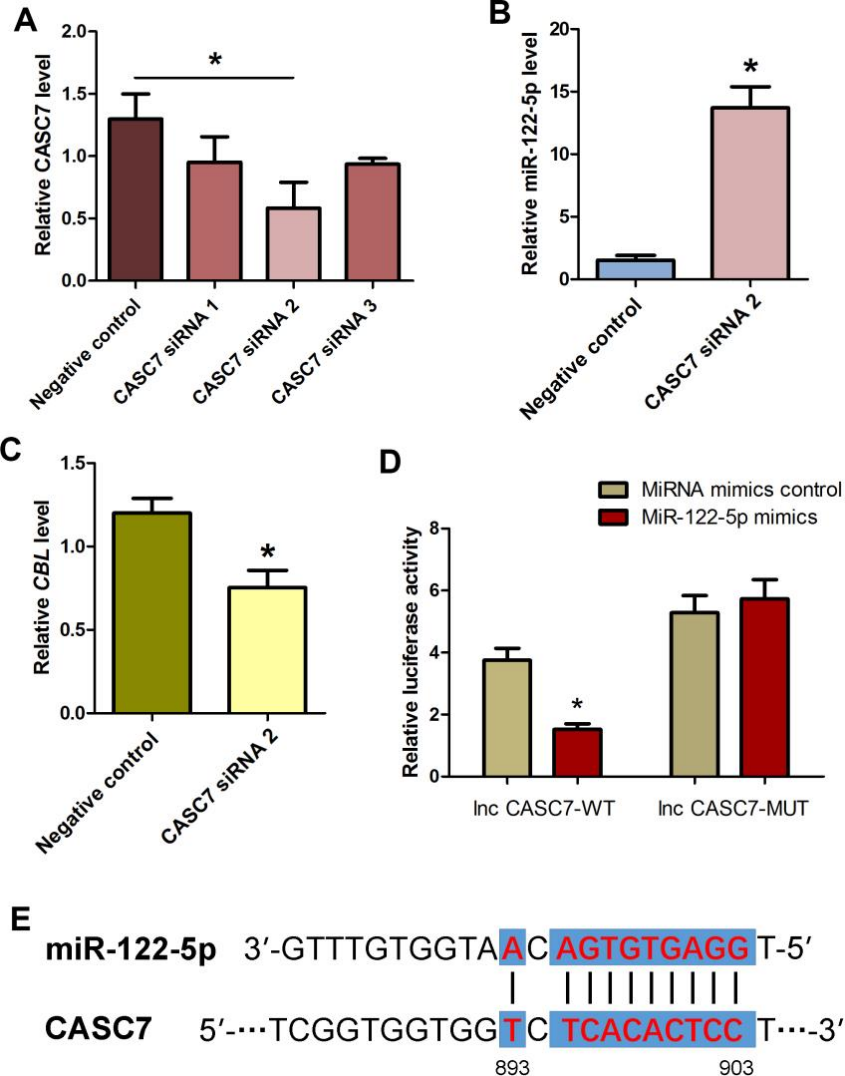
**Influence of CASC7 silencing on level of MiRNA-122-5p and CBL as well as identification and verification of the direct binding of MiRNA-122-5p and CASC7 in human SSC line.** (**A**) Real-time qPCR showed changes of CASC7 by CASC7 siRNA 1, CASC7 siRNA 2, and CASC7 siRNA 3 in human SSC line. * indicated statistically significant differences (P< 0.05) of CASC7 siRNA 1-, 2-, and 3-treated human SSC line compared with the control siRNA. (**B**) Real-time qPCR demonstrated miRNA-122-5p expression changes in human SSC line at day 2 after transfection of CASC7 siRNA 2 and the control siRNA.* denoted statistically significant differences (p<0.05) between CASC7 siRNA 2-treated cells and the control siRNA.(**C**) Real-time qPCR showed the change of *CBL* in human SSC line at day 2 after transfection of CASC7 siRNA 2 and the control siRNA.* showed statistically significant differences (p< 0.05) between CASC7 siRNA 2-treated cells and the control siRNA. (**D**) Validation of the direct binding of miRNA-122-5p to wild type CASC7 and the mutated CASC7 by dual luciferase reporter assays. * indicated statistically significant differences (p< 0.05) in human SSC line between miRNA-122-5p mimics and miRNA mimics control. (**E**) The sequences of miRNA-122-5p complement with CASC7.

It has been reported that lncRNAs communicate with mRNAs [43–45], circular RNAs [46] and miRNAs [19, 47–50], and they act as endogenous miRNA sponges, ceRNAs or the targets of miRNAs. To explore whether CASC7 communicates with miRNAs in human SSC line, we investigated the influence of lncRNA CASC7 on the level of miRNA-122-5p and *CBL* in human SSC line. Three pairs of CASC7 siRNAs (i.e., CASC7 siRNA 1, CASC7 siRNA 2, and CASC7 siRNA 3) with different binding sites were employed. Real-time qPCR demonstrated that CASC7 siRNA 2 assumed the highest potential for silencing CASC7 among three CASC7 siRNAs (Figure 7A). After CASC7 knockdown by CASC7 siRNA 2, the level of miRNA-122-5p was upregulated (Figure 7B), whereas *CBL* transcript was decreased (Figure 7C). We also constructed luciferase reporter constructs containing either the wild-type (WT) binding sequence of CASC7 RNA or the mutant forms of the seed sites into the vector. Co-transfection of the miRNA-122-5p mimics and the WT dual-luciferase reporters into 293 T cells decreased about 50% of the luciferase activity (Figure 7D), while there was no significant change in mutation of the seed sequences compared to each control groups (Figure 7D). The seed site for miRNA-122-5p in the CASC7 RNA sequence was illustrated in Figure 7E. Thus, we have revealed a network of miRNA-122-5p-CBL axis that lncRNA CASC7 competes with the miRNA-122-5p and consequently suppresses the inhibition of *CBL*.

## DISCUSSION

NOA is a multifactorial disorder that is caused by genetic, epigenetic, and environmental factors [51, 52]. Much progress has been made in revealing the molecular mechanisms underlying the spermatogenesis in rodents; however, epigenetic regulators of male germ cell development in humans remains largely unclear. Recently, we have found that miRNA-663a stimulates the proliferation and inhibits the early apoptosis of human SSCs [40]. In addition, we have revealed differentially expressed miRNAs in human spermatogonia between OA and NOA patients [41]. In this study, miRNA-122-5p was found to be expressed at a higher level in human spermatogonia of OA patients compared to NOA patients.

MiRNA-122-5p has been reported as an important modulator in diverse cell types and tissues. IL-22-induces miRNA-122-5p which promotes keratinocyte proliferation by decreasing the expression of Spry2 in the pathogenesis of psoriasis [53]. Bone marrow mesenchymal stem cells-derived exosomes carrying miRNA-122-5p reduces Sprouty2 and enhances the activity of receptor tyrosine kinase (RTK), which controls the proliferation and differentiation of rabbit osteoblasts [54]. MiRNA-122-5p stimulates the aggression and epithelial-mesenchymal transition (EMT) in triple-negative breast cancer by negative regulation of CHMP3 [55]. Since the suppression of miRNA-122-5p significantly reduces AFP level and the proliferation in AFP-producing gastric cancer (AFPGC), miRNA-122-5p has been regarded as a potential therapeutic target in AFPGC [56]. The level of miRNA-122-5p in the sperm of patients with oligospermia semen is lower than normal sperm, and thus the deficiency of miRNA-122-5p might affect the proliferation and differentiation of male germ cells, leading to a decrease in the sperm density [57]. Knockdown of ANRIL elevates the level of miRNA-122-5p and suppresses cell proliferation, metastasis and invasion in hepatocellular carcinoma (HCC) [58]. The increse in the expression of miRNA-122-5p suppresses the proliferation, migration and invasion of glioma [59], HCC [60], cervical cancer [61], gastric cancer cells [62], and nasopharyngeal carcinoma [63]. In the present study, we have demonstrated that miRNA-122-5p stimulates the proliferation and DNA synthesis and inhibits the early apoptosis of human SSCs.

Characterized by the length of C termini and capability to work as adaptors, the highly conserved ubiquitin ligases CBL families include three mammalian homologs, namely CBL (also known as c-CBL or CBL2), CBL-b and CBL-c (CBL-3). CBL is known as an E3 ubiquitin ligase, and it promotes the degradation of proteins associated with cell growth and migration [64–66]. The main function of CBL is derived from ubiquitination of active receptor tyrosine kinases (RTKs), which results in negative regulation of their signaling and directs them towards lysosomes or degradation [67]. CBL is a tumor suppressor in the pathogenesis of human cancers, and it plays roles in melanoma cell proliferation, migration and invasion [68]. Moreover, CBL restricts myeloid proliferation of human AML1-ETO-induced leukemia [69]. Regulated by Tyr371 phosphorylation, CBL inhibits the tumorigenesis of colorectal cancer via targeting Wnt/β-catenin [70]. However, mutant CBL works as oncogene in myeloid leukemia [71] and myeloproliferative neoplasms [72]. Predicted by bioinformatics and further verified by our real-time qPCR, Western blots and dual-luciferase reporter analyses, we have demonstrated that CBL is a direct target of miRNA-122-5p in human SSCs. Notably, we found that CBL knockdown by directly binding of miRNA-122-5p enhances the proliferation and DNA synthesis and inhibits the early rather than the late apoptosis of human SSC line.

Recent studies suggest that lncRNAs are highly diverse in regulating gene expression. Nevertheless, the molecular mechanisms of lncRNAs in human SSCs remain to be explored. LncRNAs possibly interact with miRNAs by acting as endogenous sponges or competing ceRNAs and further influence the expression of target genes. However, the interactions between miRNAs and lncRNAs in human SSCs remain unknown. In this study, we have obtained the differentially expressed lncRNAs by comparing and analyzing the profiles of lncRNAs in human spermatogonia between OA and NOA patients, and importantly, we found *CASC7* as the most significantly deficient lncRNA in human spermatogonia of OA patients. CASC7 inhibits the proliferation and migration of colon cancer cells via miR-21/ING3 axis [73], and it represses the proliferation of glioma cells via negatively regulating Wnt/β-catenin signaling pathway [74]. Conversely, upregulation of lncRNA CASC7 reduces the apoptosis of neuronal cells in spinal cord ischemia-reperfusion injury rats [75] and inhibits the myocardial apoptosis in myocardial ischemia-reperfusion rats by interacting with miRNA-21 [76]. As determined by real-time qPCR and dual-luciferase reporter analyses, we have revealed the negative correlation between CASC7 and miRNA-122-5p, and knockdown of CASC7 stimulates the expression of miRNA-122-5p and consequently decreases the level of *CBL*. Collectively, CASC7 is directly and negatively interact with miRNA-122-5p and may neutralize the inhibition effect of miRNA-122-5p on CBL in human SSCs.

In summary, we have demonstrated that miRNA-122-5p is expressed at a higher level in human spermatogonia in OA patients than in NOA patients. We revealed that miRNA-122-5p stimulates the proliferation and DNA synthesis and inhibits the early but not the late apoptosis of human SSCs via directly targeting CBL. LncRNA CASC7 is deficient in human spermatogonia in OA patients compared to NOA patients, and it functions as a miRNA-122-5p decoy that competes with the expression of miRNA-122-5p and further counteracts the inhibition effect of miRNA-122-5p on its target CBL. This study thus provides new lncRNA-miRNA mechanisms in human SSCs, and it adds new epigenetic regulator of human SSCs and provides novel targets for reproductive and regenerative medicine.

## MATERIALS AND METHODS

### Obtaining testicular tissues from OA and NOA patients

Testicular tissues in this study were obtained from OA and NOA patients with microdissection of testicular biopsy at Ren Ji Hospital affiliated with Shanghai Jiao Tong University School of Medicine. OA patients had normal spermatogenesis, whereas NOA patients with hypospermatogenesis and germ cell mature arrest were verified by histological analysis. The consents of testicular biopsies for research only were obtained from the donors.

### Separation of human spermatogonia from testicular tissues by STA-PUT velocity sedimentation

Testicular biopsies were washed three times with DMEM/F12 containing 1% penicillin and streptomycin (Gibco). Human male germ cells were isolated from testicular tissues via a two-step enzymatic digestion and followed by differential plating [77]. STA-PUT with 0.5%-4% BSA gradient and velocity sedimentation was utilized to separate human spermatogonia from other germ cells based upon their sizes, mass, and gravities [40]. Cellular fractions of similar size and morphological features with human spermatogonia were pooled together for subsequent analyses.

### Real-time qPCR

Total RNA was extracted from human spermatogonia and human SSC line by the RNAiso Plus reagent (Takara, Kusatsu, Japan). Concentrations of total RNA were measured by Nanodrop (Thermo Scientific, MA, USA), and RNA with the A_260_/A_280_ ratio of 1.9-2.0 was used for real-time qPCR. Reverse transcription (RT) was conducted using the First Strand cDNA Synthesis Kit (Thermo Scientific, USA), and PCR of the cDNA was performed pursuant to the method [78]. For miRNA real-time PCR, RT reaction was done using TransScript miRNA First-Strand cDNA Synthesis SuperMix Kit (Transgene). Each RT reaction comprised 100 ng RNA, 1 μl miRNA RT Enzyme Mix, 10 μl 2x TS miRNA Reaction Mix, and RNase-free water. Reactions were performed in a Veriti 96-Well Thermal Cycler (Applied Biosystems) for 60 min at 37° C and followed by heat inactivation of RT for 5 seconds at 85° C. RT reaction mix was diluted by 5 times in nuclease-free water and held at -20° C. Primer sequences of miRNAs and mRNAs for real-time qPCR were listed in Table 1. Real-time PCR was performed in triplicate using Power SYBR Green PCR Master Mix (Applied Biosystems, Woolston Warrington, UK) and a 7500 Fast Real-Time PCR System (Applied Biosystems, Carlsbad, CA, USA). Melting curve analysis was conducted to validate the specific generation of the expected PCR products. The expression levels of miRNAs were normalized to U6, while the mRNA levels were normalized to *GAPDH*. Relative levels of genes and miRNAs were calculated using the 2^-ΔΔCt^ method [79].

**Table 1:** The primer sequences for miRNAs, genes, and lncRNAs.

**Name**	**Primer sequences (5’ to 3’)**		**Tm (° C)**
Has-miR-122-5p	TGGAGTGTGACAATGGTGTTTG		60
Has-miR-373-3p	GAAGTGCTTCGATTTTGGGGTGT		60
Has-miR-100-5p	AACCCGTAGATCCGAACTTGTG		60
Has-miR-145-3p	TGAGATGAAGCACTGTAGCTC		60
U6	CGCTTCGGCAGCACATATAC		60
*ZNF668*	Forward	CGCCCATGAAGGGGTGAAG	60
	Reverse	ACGAACGTCTTGTCACATTGC	
*PLAG1*	Forward	ATCAACTCCATACACACGACC	60
	Reverse	AGCTTGGTATTGTAGTTCTTGCC	
*MED2D*	Forward	CCAGCGAATCACCGACGAG	60
	Reverse	GCAGTCACATAGCACGCTC	
*FOXP1*	Forward	ATGATGCAAGAATCTGGGACTG	60
	Reverse	AGCTGGTTGTTTGTCATTCCTC	
*CBL*	Forward	TGGTGCGGTTGTGTCAGAAC	60
	Reverse	GGTAGGTATCTGGTAGCAGGTC	
*XIST*	Forward	TCCAGTTCTGTCGCAGTGTTCAAG	60
	Reverse	GCAAGACCTTCAGCCGCCATC	
*CDKN2B-AS1*	Forward	ACAGCTCACTGCAACCTTGAACTC	60
	Reverse	GCACTGTGTCCATAGCACCTTCC	
*MAL2*	Forward	ATTGGAAGCAGCAGCCACATCC	60
	Reverse	GGAGTGTTACGGTCGCCATCTTC	
*CASC7*	Forward	AACATGGTCTCTTGGTGCCTGATG	60
	Reverse	CCACGGTAAGCGACGAGGAATC	
*GAPDH*	Forward	CAGGAGGCATTGCTGATGAT	60
	Reverse	GAAGGCTGGGGCTCATTT	

### RNA deep sequencing

RNA deep sequencing was conducted pursuant to the method [42], and an Illumina HiSeq 2000 sequencer was utilized for sequencing of miRNA libraries. RNA deep sequencing data were deposited in the Sequence Read Archive (accession no. SRP045287) [42].

### Human SSC line and cell culture

Human SSC was established previously by our team [80]. This cell line expresses a number of human male germ cell and SSC hallmarks, and it has been identified as human SSCs [40, 80]. Human SSC line was cultured in DMEM/F12 with 10% fetal bovine serum (FBS) (Life Technologies Inc., Grand Island, NY, USA) and 100 U/ml penicillin and 100 mg/ml streptomycin (Life Technologies Inc.) at 37° C in 5% CO_2_. Cells were passaged every 2-3 days using 0.05% trypsin (Invitrogen) and 0.53 mM EDTA (Invitrogen).

### Transfection of siRNAs and miRNAs and infection of lentivirus

SiRNAs, miRNA mimics and inhibitors were purchased from GenePharma (Shanghai, China). The oligonucleotides of miRNA mimics, inhibitors and siRNAs were shown in Table 2. Human SSC line was seeded at 1 x 10^5^/cm^2^ density and cultured in DMEM/F12 with 10% FBS overnight.

**Table 2:** The sequences for miRNA-122-5p mimics, miR122-5p inhibitor, and siRNA oligonucleotides against CBL and CASC7.

**MiRNAs and siRNAs**	**Sequence (5’ to 3’)**	
Hsa-miRNA-122-5p mimics	Sense	UGGAGUGUGACAAUGGUGUUUG
	Antisense	AACACCAUUGUCACACUCCAUU
MiRNA mimics control	Sense	UUCUCCGAACGUGUCACGUTT
	Antisense	ACGUGACACGUUCGGAGAATT
Hsa-miRNA-122-5p inhibitor	CAAACACCAUUGUCACACUCCA	
MiRNA inhibitor control	CAGUACUUUUGUGUAGUACAA	
CBL siRNA1	Sense	GCCUGAUUGGGCUCAUGAATT
	Antisense	UUCAUGAGCCCAAUCAGGCTT
CBL siRNA2	Sense	GGGAACAUUCUCCAGACAATT
	Antisense	UUGUCUGGAGAAUGUUCCCTT
CBLsiRNA3	Sense	GGACACCUCAUGUGCACAUTT
	Antisense	AUGUGCACAUGAGGUGUCCTT
CASC7 siRNA 1	Sense	GCGUUACACGAUGCACUUUTT
	Antisense	AAAGUGCAUCGUGUAACGCTT
CASC7 siRNA 2	Sense	GCAAGAAGAGAUUAGCAAATT
	Antisense	UUUGCUAAUCUCUUCUUGCTT
CASC7 siRNA 3	Sense	GGUACCACCUGGUGGAUAATT
	Antisense	UUAUCCACCAGGUGGUACCTT
Control siRNA	Sense	UUCUCCGAACGUGUCACGUTT
	Antisense	ACGUGACACGUUCGGAGAATT

Transfection of miRNA mimics or inhibitor and siRNAs was performed using lipofectamine 3000 transfection agent (Life Technologies, Carlsbad, CA, USA). After 48 hr of culture, cells were harvested for subsequent analyses.

The lentivirus, namely PGMLV-CMV-MCS-EF1-ZsGreen1, was purchased from Jiman Biotechnology CO., LTD (Shanghai, China). Human SSC line was plated at a density of 5 × 10^5^ cells in 100 mm plates, and these cells were cultured with DMEM/F12 and 10% FBS overnight. Culture medium was changed with fresh DMEM/F12 containing 10^8^ TU/ml PGMLV-CMV-MCS-EF1-ZsGreen1 and 10 μg/ml polybrene, and the cells were incubated at 37° C in 5% CO_2_ overnight. After 24 hr of culture, the medium was changed with fresh DMEM/F12 and 10% FBS, and the expression of ZsGreen1 was detected under a fluorescence microscope (Nikon Eclipse Ti-S, Nikon Corporation, Tokyo, Japan). The infected cells were cultured and expanded in DMEM/F12 medium supplemented with 10% FBS and 1% antibiotic containing penicillin and streptomycin (Gibco).

### CCK-8 assay

Human SSC line was seeded at a density of 2,000 cells/well in 96-well microtiter plates in DMEM/F12 with 1% FBS. After 5 days of culture, the proliferation potential of human SSC line was examined by CCK-8 assay (Dojin Laboratories, Kumamoto, Japan) according to the manufacturer’s instruction.

### Western blots

Human SSC line with treatment of lentivirus, miRNA oligonucleotides or siRNAs was lysed with RIPA buffer (Santa Cruz Biotechnology). After 30 min of lysis on ice, cell lysates were cleared by centrifugation at 12,000 × *g* for 20 min, and the concentrations of proteins were measured by BCA kit (Dingguo, China). Thirty micrograms of cell lysate from each cell sample were used for SDS-PAGE (Bio-Rad), and Western blots were performed according to the protocol [81]. The information of primary antibodies was listed in Table 3. After extensive washes with tris-buffered saline and Tween 20, the blots were detected by chemiluminescence (Chemi-Doc XRS, Bio-Rad, Hercules, CA, USA).

**Table 3:** Primary antibodies’ information for Western blots.

**Antibodies**	**Vendors**	**Sources**	**Working dilutions**
PCNA	CST	Rabbit	1:1000
CBL	R&D	Mouse	1:1000
ACTB	Proteintech	Mouse	1:2000

### EDU incorporation assay

Human SSC line was seeded at a density of 5,000 cells/well in a 96-well plate, and these cells were treated with lentivirus, miRNA oligonucleotides or siRNAs and followed by 50 mM EDU (RiboBio, Guangzhou, China). After 12 hr of culture, the cells were washed with DMEM and fixed with 4% PFA. Cells were neutralized with 2 mg/ml glycine and permeabilized in 0.5% Triton X-100 for 10 min at room temperature. EDU immunostaining was performed with Apollo staining reaction buffer. The nuclei of cells were stained with Hoechst 33342, and the EDU-positive cells were counted from at least 500 cells under fluorescence microscopy (Nikon, Tokyo, Japan).

### Annexin V and PI staining and flow cytometry analysis

The early and late apoptosis in human SSC line was measured using the APC Annexin V and PI apoptosis detection kit and flow cytometry pursuant to the manufacturer’s instruction. Human SSC line was seeded at a density of 5 x 10^4^ cells/well in 12-well plates, and cells were collected by centrifuging at 1,000 rpm for 5 min at day 2 after infection or day 3 after transfection. The cells were simultaneously stained with Annexin V-FITC (green fluorescence) and the non-vital dye PI (red fluorescence), which identified early apoptotic cells (FITC^+^PI^-^) and late apoptotic cells (FITC^+^PI^+^).

### Dual luciferase assays

Human SSC line was seeded in a 48-well culture plate and cultured at 37° C in 5% CO_2_ for 24 hr. MiRNA mimics were first transfected to the cells using lipofectamine 3000 transfection agent (Life Technologies, Carlsbad, CA, USA). Ten hours later, 500 ng plasmids with the target sequences, Firefly Luciferase (reporter), and Renilla Luciferase (internal control) (pMIR-GIO, Genecreate, Wuhan, China) were transfected by lipofectamine 3000 reagent (Life Technologies, Carlsbad, CA, USA). Cells were lysed after 48 hr of transfection, and luciferase activity was measured using the tube luminometer (Berthold, Germany). Data were normalized to miRNA mimic control-transfected cells.

### LncRNA microarray assays

Total RNA was isolated from human spermatogonia of OA and NOA patients using RNAiso Plus reagent (Takara, Kusatsu, Japan). DNase I was used to remove potential genomic DNA contamination. The quality of Total RNAs was determined by the NanoDrop ND-2000 (Thermo Scientific), and RNA integrity was assessed using Agilent Bioanalyzer 2100 (Agilent Technologies). The sample labeling, microarray hybridization and washing were performed based upon the manufacturer’s standard protocols. Briefly, total RNA was transcribed to double strand cDNAs and then synthesized cRNAs. Next, 2^nd^ cycle cDNAs were synthesized from cRNAs. Fragmentation and biotin labeling and the 2^nd^ cycle cDNAs were hybridized onto the Affymetrix human lncRNA arrays. After washing and staining, the arrays were scanned by the Affymetrix Scanner 3000 (Affymetrix). Raw data were generated from Affymetrix GeneChip Command Console (version 4.0, Affymetrix) software, and 63,542 lncRNAs were tested using these arrays. Data analysis of gene expression profiling was processed by Genespring software (version 14.9; Agilent Technologies). Differentially expressed lncRNAs were calculated by pairwise combination and error-weighted average. The threshold set for upregulated and downregulated lncRNAs was with a fold change≥2.0 and p value < 0.05, and the false discovery rate (FDR) was adjusted p-values (Benjamini-Hochberg) <0.05.

### Statistical analysis

All data were presented as mean ± SEM from at least three independent experiments and analyzed by t test using Prism (version 5, GraphPad), and p value< 0.05 was considered statistically significant.

## References

[1] Oatley JM, Brinster RL. Regulation of spermatogonial stem cell self-renewal in mammals. Annu Rev Cell Dev Biol. 2008; 24:263–86. 10.1146/annurev.cellbio.24.110707.175355 18588486 PMC4066667

[2] Conrad S, Renninger M, Hennenlotter J, Wiesner T, Just L, Bonin M, Aicher W, Bühring HJ, Mattheus U, Mack A, Wagner HJ, Minger S, Matzkies M, et al. Generation of pluripotent stem cells from adult human testis. Nature. 2008; 456:344–49. 10.1038/nature07404 18849962

[3] Zhang Z, Gong Y, Guo Y, Hai Y, Yang H, Yang S, Liu Y, Ma M, Liu L, Li Z, Gao WQ, He Z. Direct transdifferentiation of spermatogonial stem cells to morphological, phenotypic and functional hepatocyte-like cells via the ERK1/2 and Smad2/3 signaling pathways and the inactivation of cyclin A, cyclin B and cyclin E. Cell Commun Signal. 2013; 11:67. 10.1186/1478-811X-11-67 24047406 PMC3848919

[4] Chen Z, Niu M, Sun M, Yuan Q, Yao C, Hou J, Wang H, Wen L, Fu H, Zhou F, Li Z, He Z. Transdifferentiation of human male germline stem cells to hepatocytes in vivo via the transplantation under renal capsules. Oncotarget. 2017; 8:14576–92. 10.18632/oncotarget.14713 28107194 PMC5362427

[5] Simon L, Ekman GC, Kostereva N, Zhang Z, Hess RA, Hofmann MC, Cooke PS. Direct transdifferentiation of stem/progenitor spermatogonia into reproductive and nonreproductive tissues of all germ layers. Stem Cells. 2009; 27:1666–75. 10.1002/stem.93 19544441 PMC2904909

[6] Phillips BT, Gassei K, Orwig KE. Spermatogonial stem cell regulation and spermatogenesis. Philos Trans R Soc Lond B Biol Sci. 2010; 365:1663–78. 10.1098/rstb.2010.0026 20403877 PMC2871929

[7] Reinke V, Smith HE, Nance J, Wang J, Van Doren C, Begley R, Jones SJ, Davis EB, Scherer S, Ward S, Kim SK. A global profile of germline gene expression in C. Elegans. Mol Cell. 2000; 6:605–16. 10.1016/s1097-2765(00)00059-9 11030340

[8] Meng X, Lindahl M, Hyvönen ME, Parvinen M, de Rooij DG, Hess MW, Raatikainen-Ahokas A, Sainio K, Rauvala H, Lakso M, Pichel JG, Westphal H, Saarma M, Sariola H. Regulation of cell fate decision of undifferentiated spermatogonia by GDNF. Science. 2000; 287:1489–93. 10.1126/science.287.5457.1489 10688798

[9] Wang RS, Yeh S, Tzeng CR, Chang C. Androgen receptor roles in spermatogenesis and fertility: lessons from testicular cell-specific androgen receptor knockout mice. Endocr Rev. 2009; 30:119–32. 10.1210/er.2008-0025 19176467 PMC2662628

[10] Hai Y, Hou J, Liu Y, Liu Y, Yang H, Li Z, He Z. The roles and regulation of sertoli cells in fate determinations of spermatogonial stem cells and spermatogenesis. Semin Cell Dev Biol. 2014; 29:66–75. 10.1016/j.semcdb.2014.04.007 24718316

[11] Reinhart BJ, Slack FJ, Basson M, Pasquinelli AE, Bettinger JC, Rougvie AE, Horvitz HR, Ruvkun G. The 21-nucleotide let-7 RNA regulates developmental timing in caenorhabditis elegans. Nature. 2000; 403:901–06. 10.1038/35002607 10706289

[12] Lee RC, Feinbaum RL, Ambros V. The C. Elegans heterochronic gene lin-4 encodes small RNAs with antisense complementarity to lin-14. Cell. 1993; 75:843–54. 10.1016/0092-8674(93)90529-y 8252621

[13] Guttman M, Amit I, Garber M, French C, Lin MF, Feldser D, Huarte M, Zuk O, Carey BW, Cassady JP, Cabili MN, Jaenisch R, Mikkelsen TS, et al. Chromatin signature reveals over a thousand highly conserved large non-coding RNAs in mammals. Nature. 2009; 458:223–27. 10.1038/nature07672 19182780 PMC2754849

[14] Guttman M, Rinn JL. Modular regulatory principles of large non-coding RNAs. Nature. 2012; 482:339–46. 10.1038/nature10887 22337053 PMC4197003

[15] Kawasaki H, Taira K. Retraction: Hes1 is a target of microRNA-23 during retinoic-acid-induced neuronal differentiation of NT2 cells. Nature. 2003; 426:100. 10.1038/nature02141 14603326

[16] Faghihi MA, Modarresi F, Khalil AM, Wood DE, Sahagan BG, Morgan TE, Finch CE, St Laurent G 3rd, Kenny PJ, Wahlestedt C. Expression of a noncoding RNA is elevated in Alzheimer’s disease and drives rapid feed-forward regulation of beta-secretase. Nat Med. 2008; 14:723–30. 10.1038/nm1784 18587408 PMC2826895

[17] Faghihi MA, Zhang M, Huang J, Modarresi F, Van der Brug MP, Nalls MA, Cookson MR, St-Laurent G 3rd, Wahlestedt C. Evidence for natural antisense transcript-mediated inhibition of microRNA function. Genome Biol. 2010; 11:R56. 10.1186/gb-2010-11-5-r56 20507594 PMC2898074

[18] Wang J, Liu X, Wu H, Ni P, Gu Z, Qiao Y, Chen N, Sun F, Fan Q. CREB up-regulates long non-coding RNA, HULC expression through interaction with microRNA-372 in liver cancer. Nucleic Acids Res. 2010; 38:5366–83. 10.1093/nar/gkq285 20423907 PMC2938198

[19] Cesana M, Cacchiarelli D, Legnini I, Santini T, Sthandier O, Chinappi M, Tramontano A, Bozzoni I. A long noncoding RNA controls muscle differentiation by functioning as a competing endogenous RNA. Cell. 2011; 147:358–69. 10.1016/j.cell.2011.09.028 22000014 PMC3234495

[20] Yang QE, Racicot KE, Kaucher AV, Oatley MJ, Oatley JM. MicroRNAs 221 and 222 regulate the undifferentiated state in mammalian male germ cells. Development. 2013; 140:280–90. 10.1242/dev.087403 23221369 PMC3597206

[21] He Z, Jiang J, Kokkinaki M, Tang L, Zeng W, Gallicano I, Dobrinski I, Dym M. MiRNA-20 and mirna-106a regulate spermatogonial stem cell renewal at the post-transcriptional level via targeting STAT3 and Ccnd1. Stem Cells. 2013; 31:2205–17. 10.1002/stem.1474 23836497 PMC3859454

[22] Chen J, Cai T, Zheng C, Lin X, Wang G, Liao S, Wang X, Gan H, Zhang D, Hu X, Wang S, Li Z, Feng Y, et al. MicroRNA-202 maintains spermatogonial stem cells by inhibiting cell cycle regulators and RNA binding proteins. Nucleic Acids Res. 2017; 45:4142–57. 10.1093/nar/gkw1287 27998933 PMC5397178

[23] Huang YL, Huang GY, Lv J, Pan LN, Luo X, Shen J. miR-100 promotes the proliferation of spermatogonial stem cells via regulating Stat3. Mol Reprod Dev. 2017; 84:693–701. 10.1002/mrd.22843 28569396

[24] Li J, Liu X, Hu X, Tian GG, Ma W, Pei X, Wang Y, Wu J. MicroRNA-10b regulates the renewal of spermatogonial stem cells through kruppel-like factor 4. Cell Biochem Funct. 2017; 35:184–91. 10.1002/cbf.3263 28436141

[25] Liu SS, Maguire EM, Bai YS, Huang L, Liu Y, Xu L, Fauzi I, Zhang SQ, Xiao Q, Ma NF. A novel regulatory axis, CHD1L-MicroRNA 486-matrix metalloproteinase 2, controls spermatogonial stem cell properties. Mol Cell Biol. 2019; 39:e00357–18. 10.1128/MCB.00357-18 30455250 PMC6362313

[26] Wang Y, Li X, Gong X, Zhao Y, Wu J. MicroRNA-322 regulates self-renewal of mouse spermatogonial stem cells through Rassf8. Int J Biol Sci. 2019; 15:857–69. 10.7150/ijbs.30611 30906216 PMC6429012

[27] Houbaviy HB, Murray MF, Sharp PA. Embryonic stem cell-specific MicroRNAs. Dev Cell. 2003; 5:351–58. 10.1016/s1534-5807(03)00227-2 12919684

[28] Hayashi K, Chuva de Sousa Lopes SM, Kaneda M, Tang F, Hajkova P, Lao K, O’Carroll D, Das PP, Tarakhovsky A, Miska EA, Surani MA. MicroRNA biogenesis is required for mouse primordial germ cell development and spermatogenesis. PLoS One. 2008; 3:e1738. 10.1371/journal.pone.0001738 18320056 PMC2254191

[29] Tong MH, Mitchell D, Evanoff R, Griswold MD. Expression of Mirlet7 family microRNAs in response to retinoic acid-induced spermatogonial differentiation in mice. Biol Reprod. 2011; 85:189–97. 10.1095/biolreprod.110.089458 21430230 PMC3123386

[30] Tong MH, Mitchell DA, McGowan SD, Evanoff R, Griswold MD. Two miRNA clusters, mir-17-92 (Mirc1) and Mir-106b-25 (Mirc3), are involved in the regulation of spermatogonial differentiation in mice. Biol Reprod. 2012; 86:72. 10.1095/biolreprod.111.096313 22116806 PMC3316268

[31] Huszar JM, Payne CJ. MicroRNA 146 (Mir146) modulates spermatogonial differentiation by retinoic acid in mice. Biol Reprod. 2013; 88:15. 10.1095/biolreprod.112.103747 23221399 PMC4434937

[32] Yu M, Mu H, Niu Z, Chu Z, Zhu H, Hua J. miR-34c enhances mouse spermatogonial stem cells differentiation by targeting Nanos2. J Cell Biochem. 2014; 115:232–42. 10.1002/jcb.24655 24038201

[33] Xie R, Lin X, Du T, Xu K, Shen H, Wei F, Hao W, Lin T, Lin X, Qin Y, Wang H, Chen L, Yang S, et al. Targeted disruption of miR-17-92 impairs mouse spermatogenesis by activating mTOR signaling pathway. Medicine (Baltimore). 2016; 95:e2713. 10.1097/MD.0000000000002713 26886608 PMC4998608

[34] Guttman M, Donaghey J, Carey BW, Garber M, Grenier JK, Munson G, Young G, Lucas AB, Ach R, Bruhn L, Yang X, Amit I, Meissner A, et al. lincRNAs act in the circuitry controlling pluripotency and differentiation. Nature. 2011; 477:295–300. 10.1038/nature10398 21874018 PMC3175327

[35] Wang Y, Xu Z, Jiang J, Xu C, Kang J, Xiao L, Wu M, Xiong J, Guo X, Liu H. Endogenous miRNA sponge lincRNA-RoR regulates Oct4, Nanog, and Sox2 in human embryonic stem cell self-renewal. Dev Cell. 2013; 25:69–80. 10.1016/j.devcel.2013.03.002 23541921

[36] Li L, Wang M, Wang M, Wu X, Geng L, Xue Y, Wei X, Jia Y, Wu X. A long non-coding RNA interacts with Gfra1 and maintains survival of mouse spermatogonial stem cells. Cell Death Dis. 2016; 7:e2140. 10.1038/cddis.2016.24 26962690 PMC4823932

[37] Hu K, Zhang J, Liang M. LncRNA AK015322 promotes proliferation of spermatogonial stem cell C18-4 by acting as a decoy for microRNA-19b-3p. In Vitro Cell Dev Biol Anim. 2017; 53:277–84. 10.1007/s11626-016-0102-5 27822884

[38] Kataruka S, Akhade VS, Kayyar B, Rao MR. Mrhl long noncoding RNA mediates meiotic commitment of mouse spermatogonial cells by regulating Sox8 expression. Mol Cell Biol. 2017; 37:e00632–16. 10.1128/MCB.00632-16 28461394 PMC5492173

[39] Hu K, Li L, Liao Y, Liang M. LncRNA Gm2044 highly expresses in spermatocyte and inhibits Utf1 translation by interacting with Utf1 mRNA. Genes Genomics. 2018; 40:781–87. 10.1007/s13258-018-0690-4 29934815

[40] Zhou F, Yuan Q, Zhang W, Niu M, Fu H, Qiu Q, Mao G, Wang H, Wen L, Wang H, Lu M, Li Z, He Z. MiR-663a stimulates proliferation and suppresses early apoptosis of human spermatogonial stem cells by targeting NFIX and regulating cell cycle. Mol Ther Nucleic Acids. 2018; 12:319–36. 10.1016/j.omtn.2018.05.015 30195770 PMC6037887

[41] Liu Y, Niu M, Yao C, Hai Y, Yuan Q, Liu Y, Guo Y, Li Z, He Z. Fractionation of human spermatogenic cells using STA-PUT gravity sedimentation and their miRNA profiling. Sci Rep. 2015; 5:8084. 10.1038/srep08084 25634318 PMC5155379

[42] Yao C, Yuan Q, Niu M, Fu H, Zhou F, Zhang W, Wang H, Wen L, Wu L, Li Z, He Z. Distinct expression profiles and novel targets of MicroRNAs in human spermatogonia, pachytene spermatocytes, and round spermatids between OA patients and NOA patients. Mol Ther Nucleic Acids. 2017; 9:182–94. 10.1016/j.omtn.2017.09.007 29246297 PMC5645173

[43] Zhao J, Sun BK, Erwin JA, Song JJ, Lee JT. Polycomb proteins targeted by a short repeat RNA to the mouse X chromosome. Science. 2008; 322:750–56. 10.1126/science.1163045 18974356 PMC2748911

[44] Khalil AM, Guttman M, Huarte M, Garber M, Raj A, Rivea Morales D, Thomas K, Presser A, Bernstein BE, van Oudenaarden A, Regev A, Lander ES, Rinn JL. Many human large intergenic noncoding RNAs associate with chromatin-modifying complexes and affect gene expression. Proc Natl Acad Sci USA. 2009; 106:11667–72. 10.1073/pnas.0904715106 19571010 PMC2704857

[45] Werner MS, Ruthenburg AJ. Nuclear fractionation reveals thousands of chromatin-tethered noncoding RNAs adjacent to active genes. Cell Rep. 2015; 12:1089–98. 10.1016/j.celrep.2015.07.033 26257179 PMC5697714

[46] Hansen TB, Jensen TI, Clausen BH, Bramsen JB, Finsen B, Damgaard CK, Kjems J. Natural RNA circles function as efficient microRNA sponges. Nature. 2013; 495:384–88. 10.1038/nature11993 23446346

[47] Yamamura S, Imai-Sumida M, Tanaka Y, Dahiya R. Interaction and cross-talk between non-coding RNAs. Cell Mol Life Sci. 2018; 75:467–84. 10.1007/s00018-017-2626-6 28840253 PMC5765200

[48] Lü M, Tian H, Cao YX, He X, Chen L, Song X, Ping P, Huang H, Sun F. Downregulation of miR-320a/383-sponge-like long non-coding RNA NLC1-C (narcolepsy candidate-region 1 genes) is associated with male infertility and promotes testicular embryonal carcinoma cell proliferation. Cell Death Dis. 2015; 6:e1960. 10.1038/cddis.2015.267 26539909 PMC4670917

[49] Karreth FA, Pandolfi PP. ceRNA cross-talk in cancer: when ce-bling rivalries go awry. Cancer Discov. 2013; 3:1113–21. 10.1158/2159-8290.CD-13-0202 24072616 PMC3801300

[50] Cao C, Sun J, Zhang D, Guo X, Xie L, Li X, Wu D, Liu L. The long intergenic noncoding RNA UFC1, a target of MicroRNA 34a, interacts with the mRNA stabilizing protein HuR to increase levels of β-catenin in HCC cells. Gastroenterology. 2015; 148:415–26.e18. 10.1053/j.gastro.2014.10.012 25449213

[51] Jarow JP, Espeland MA, Lipshultz LI. Evaluation of the azoospermic patient. J Urol. 1989; 142:62–65. 10.1016/s0022-5347(17)38662-7 2499695

[52] Venkatesh T, Suresh PS, Tsutsumi R. New insights into the genetic basis of infertility. Appl Clin Genet. 2014; 7:235–43. 10.2147/TACG.S40809 25506236 PMC4259396

[53] Jiang M, Ma W, Gao Y, Jia K, Zhang Y, Liu H, Sun Q. IL-22-induced miR-122-5p promotes keratinocyte proliferation by targeting Sprouty2. Exp Dermatol. 2017; 26:368–74. 10.1111/exd.13270 27943426

[54] Liao W, Ning Y, Xu HJ, Zou WZ, Hu J, Liu XZ, Yang Y, Li ZH. BMSC-derived exosomes carrying microRNA-122-5p promote proliferation of osteoblasts in osteonecrosis of the femoral head. Clin Sci (Lond). 2019; 133:1955–75. 10.1042/CS20181064 31387936

[55] Wang Z, Wang X. miR-122-5p promotes aggression and epithelial-mesenchymal transition in triple-negative breast cancer by suppressing charged multivesicular body protein 3 through mitogen-activated protein kinase signaling. J Cell Physiol. 2020; 235:2825–35. 10.1002/jcp.29188 31541468

[56] Maruyama S, Furuya S, Shiraishi K, Shimizu H, Saito R, Akaike H, Hosomura N, Kawaguchi Y, Amemiya H, Kawaida H, Sudo M, Inoue S, Kono H, Ichikawa D. Inhibition of apoptosis by miR-122-5p in α-fetoprotein-producing gastric cancer. Oncol Rep. 2019; 41:2595–600. 10.3892/or.2019.7023 30816512

[57] Zhu M, Fei L, Li D, Chen D. Correlation analysis of miR-122-5p and occludin with sperm density in oligospermia patients’ sperm. Clin Lab. 2019; 65. 10.7754/Clin.Lab.2018.180814 30868853

[58] Ma J, Li T, Han X, Yuan H. Knockdown of LncRNA ANRIL suppresses cell proliferation, metastasis, and invasion via regulating miR-122-5p expression in hepatocellular carcinoma. J Cancer Res Clin Oncol. 2018; 144:205–14. 10.1007/s00432-017-2543-y 29127494 PMC11813335

[59] Li C, Hu G, Wei B, Wang L, Liu N. lncRNA LINC01494 promotes proliferation, migration and invasion in glioma through miR-122-5p/CCNG1 axis. Onco Targets Ther. 2019; 12:7655–62. 10.2147/OTT.S213345 31571916 PMC6756415

[60] Yang X, Sun L, Wang L, Yao B, Mo H, Yang W. LncRNA SNHG7 accelerates the proliferation, migration and invasion of hepatocellular carcinoma cells via regulating miR-122-5p and RPL4. Biomed Pharmacother. 2019; 118:109386. 10.1016/j.biopha.2019.109386 31545291

[61] Li Y, Wang H, Huang H. Long non-coding RNA MIR205HG function as a ceRNA to accelerate tumor growth and progression via sponging miR-122-5p in cervical cancer. Biochem Biophys Res Commun. 2019; 514:78–85. 10.1016/j.bbrc.2019.04.102 31023531

[62] Pei ZJ, Zhang ZG, Hu AX, Yang F, Gai Y. miR-122-5p inhibits tumor cell proliferation and induces apoptosis by targeting MYC in gastric cancer cells. Pharmazie. 2017; 72:344–47. 10.1691/ph.2017.6404 29442023

[63] Liu YH, Liu JL, Wang Z, Zhu XH, Chen XB, Wang MQ. MiR-122-5p suppresses cell proliferation, migration and invasion by targeting SATB1 in nasopharyngeal carcinoma. Eur Rev Med Pharmacol Sci. 2019; 23:622–29. 10.26355/eurrev_201901_16876 30720170

[64] Schmidt MH, Dikic I. The cbl interactome and its functions. Nat Rev Mol Cell Biol. 2005; 6:907–18. 10.1038/nrm1762 16227975

[65] Tan YH, Krishnaswamy S, Nandi S, Kanteti R, Vora S, Onel K, Hasina R, Lo FY, El-Hashani E, Cervantes G, Robinson M, Hsu HS, Kales SC, et al. CBL is frequently altered in lung cancers: its relationship to mutations in MET and EGFR tyrosine kinases. PLoS One. 2010; 5:e8972. 10.1371/journal.pone.0008972 20126411 PMC2813301

[66] Huang C. Roles of E3 ubiquitin ligases in cell adhesion and migration. Cell Adh Migr. 2010; 4:10–18. 10.4161/cam.4.1.9834 20009572 PMC2852552

[67] Liyasova MS, Ma K, Lipkowitz S. Molecular pathways: cbl proteins in tumorigenesis and antitumor immunity-opportunities for cancer treatment. Clin Cancer Res. 2015; 21:1789–94. 10.1158/1078-0432.CCR-13-2490 25477533 PMC4401614

[68] Nihal M, Wood GS. c-CBL regulates melanoma proliferation, migration, invasion and the FAK-SRC-GRB2 nexus. Oncotarget. 2016; 7:53869–80. 10.18632/oncotarget.10861 27472394 PMC5288227

[69] Goyama S, Schibler J, Gasilina A, Shrestha M, Lin S, Link KA, Chen J, Whitman SP, Bloomfield CD, Nicolet D, Assi SA, Ptasinska A, Heidenreich O, et al. UBASH3B/Sts-1-CBL axis regulates myeloid proliferation in human preleukemia induced by AML1-ETO. Leukemia. 2016; 30:728–39. 10.1038/leu.2015.275 26449661 PMC4775400

[70] Kumaradevan S, Lee SY, Richards S, Lyle C, Zhao Q, Tapan U, Jiangliu Y, Ghumman S, Walker J, Belghasem M, Arinze N, Kuhnen A, Weinberg J, et al. C-cbl expression correlates with human colorectal cancer survival and its Wnt/β-catenin suppressor function is regulated by Tyr371 phosphorylation. Am J Pathol. 2018; 188:1921–33. 10.1016/j.ajpath.2018.05.007 30029779 PMC6099425

[71] Nadeau SA, An W, Mohapatra BC, Mushtaq I, Bielecki TA, Luan H, Zutshi N, Ahmad G, Storck MD, Sanada M, Ogawa S, Band V, Band H. Structural determinants of the gain-of-function phenotype of human leukemia-associated mutant CBL oncogene. J Biol Chem. 2017; 292:3666–82. 10.1074/jbc.M116.772723 28082680 PMC5339751

[72] Aranaz P, Hurtado C, Erquiaga I, Miguéliz I, Ormazábal C, Cristobal I, García-Delgado M, Novo FJ, Vizmanos JL. CBL mutations in myeloproliferative neoplasms are also found in the gene’s proline-rich domain and in patients with the V617FJAK2. Haematologica. 2012; 97:1234–41. 10.3324/haematol.2011.052605 22315494 PMC3409822

[73] Zhang Z, Fu C, Xu Q, Wei X. Long non-coding RNA CASC7 inhibits the proliferation and migration of colon cancer cells via inhibiting microRNA-21. Biomed Pharmacother. 2017; 95:1644–53. 10.1016/j.biopha.2017.09.052 28954383

[74] Gong X, Liao X, Huang M. LncRNA CASC7 inhibits the progression of glioma via regulating Wnt/β-catenin signaling pathway. Pathol Res Pract. 2019; 215:564–70. 10.1016/j.prp.2019.01.018 30661904

[75] Liu Y, Pan L, Jiang A, Yin M. Hydrogen sulfide upregulated lncRNA CasC7 to reduce neuronal cell apoptosis in spinal cord ischemia-reperfusion injury rat. Biomed Pharmacother. 2018; 98:856–62. 10.1016/j.biopha.2017.12.079 29571256

[76] Liao B, Gao F, Lin F, Yang S, Xu Z, Dong S. LncRNA CASC7 inhibits myocardial apoptosis in myocardial ischemia-reperfusion rats by regulating MiR-21 expression. Panminerva Med. 2019. [Epub ahead of print]. 10.23736/S0031-0808.19.03728-5 31362482

[77] He Z, Kokkinaki M, Jiang J, Dobrinski I, Dym M. Isolation, characterization, and culture of human spermatogonia. Biol Reprod. 2010; 82:363–72. 10.1095/biolreprod.109.078550 19846602 PMC2809226

[78] Yang S, Ping P, Ma M, Li P, Tian R, Yang H, Liu Y, Gong Y, Zhang Z, Li Z, He Z. Generation of haploid spermatids with fertilization and development capacity from human spermatogonial stem cells of cryptorchid patients. Stem Cell Reports. 2014; 3:663–75. 10.1016/j.stemcr.2014.08.004 25358793 PMC4223697

[79] Wang H, Yuan Q, Sun M, Niu M, Wen L, Fu H, Zhou F, Chen Z, Yao C, Hou J, Shen R, Lin Q, Liu W, et al. BMP6 regulates proliferation and apoptosis of human sertoli cells via Smad2/3 and cyclin D1 pathway and DACH1 and TFAP2A activation. Sci Rep. 2017; 7:45298. 10.1038/srep45298 28387750 PMC5384448

[80] Hou J, Niu M, Liu L, Zhu Z, Wang X, Sun M, Yuan Q, Yang S, Zeng W, Liu Y, Li Z, He Z. Establishment and characterization of human germline stem cell line with unlimited proliferation potentials and no tumor formation. Sci Rep. 2015; 5:16922. 10.1038/srep16922 26585066 PMC4653657

[81] He Z, Jiang J, Hofmann MC, Dym M. Gfra1 silencing in mouse spermatogonial stem cells results in their differentiation via the inactivation of RET tyrosine kinase. Biol Reprod. 2007; 77:723–33. 10.1095/biolreprod.107.062513 17625109 PMC2911237

